# MicroRNA-199a-3p suppresses glioma cell proliferation by regulating the AKT/mTOR signaling pathway

**DOI:** 10.1007/s13277-015-3409-z

**Published:** 2015-04-09

**Authors:** Liang Shen, Chunming Sun, Yanyan Li, Xuetao Li, Ting Sun, Chuanjin Liu, Youxin Zhou, Ziwei Du

**Affiliations:** Neurosurgery and Brain and Nerve Research Laboratory, The First Affiliated Hospital of Soochow University, 188 Shizi Street, Suzhou, Jiangsu 215006 China

**Keywords:** Glioma, miR-199a-3p, mTOR, Proliferation

## Abstract

Glioma has been investigated for decades, but the prognosis remains poor because of rapid proliferation, its aggressive potential, and its resistance to chemotherapy or radiotherapy. The mammalian target of rapamycin (mTOR) is highly expressed and regulates cellular proliferation and cell growth. MicroRNAs (miRNAs) are small non-coding RNAs that regulate gene transcription and translation via up-regulating or down-regulating the levels of miRNAs. This study was conducted to explore the molecular functions of miR-199a-3p in glioma. We detected the expression of miR-199a-3p in glioma samples by quantitative PCR (qPCR). Then, we transfected the U87 and U251 cell lines with miR-199a-3p. Cellular proliferation, invasion, and apoptosis were assessed to explain the function of miR-199a-3p. PCR confirmed that the expression of miR-199a-3p was lower in glioma samples combined with normal brain tissues. The over-expression of miR-199a-3p might target mTOR and restrained cellular growth and proliferation but not invasive and apoptosis capability. Results indicated that cellular proliferation was inhibited to regulate the AKT/mTOR signaling pathway by elevating levels of miR-199a-3p. MiR-199a-3p in glioma cell lines has effects similar to the tumor suppressor gene on cellular proliferation via the AKT/mTOR signaling pathway.

## Introduction

Malignant glioma is a lethal human primary brain tumor with limited therapeutic options. Glioma is devastating due to diffuse invasion, chemical, and radiation therapy resistance [1]. The PI3K/Akt/mTOR pathway in human tumor cells is often constitutively activated [2]. Wangpaichitr et al. have demonstrated that mTOR plays a pivotal role in cisplatin resistance [3]. Moreover, cancer remains a poor prognosis, mainly attributed to the excessive activation of the PI3K-AKT/mTOR signaling pathway [4–8]. Now, with the improvement of the prognosis by targeting the mTOR, inhibitors have been used for blocking the PI3K-AKT/mTOR signaling pathway and have been preliminarily demonstrated to be an anticancer therapeutic intervention in glioblastoma [9–12].

Evidence collected confirms that microRNAs (miRNAs) are involved in the initiation, development, and prognosis of cancer, and they have multiple roles in different tissues. miRNAs are continuously being found to act in roles analogous to tumor suppressors and oncogenes [13, 14]. Among miRNAs analyzed, we found miR-199a-3p was detected to be a significantly different expression in glioma cell lines SHG139 compared with SHG139s (*P* < 0.01). Subsequently, a pre-experiment was carried out to define differential expressions in glioma samples combined with normal brain tissues. Furthermore, miR-199a-3p was a reduced expression in endometrioid adenocarcinoma, papillary thyroid carcinoma, ovarian cancer, and hepatocellular carcinoma [15–18]. In addition, thirteen nucleotides are complementary to miR-199a-3p and mTOR sequences in the miRBase database. Besides, many previous studies indicate that miR-199a-3p targets mTOR by luciferase reporter analysis [15, 17, 18], and cell proliferation is modulated through inhibiting mTOR.

## Materials and methods

### Human tissue samples

Specimens of human brain tumors for the study were collected from participants who underwent surgical removal of their brain tumors at the Department of Neurosurgery, Brain and Nerve Research Laboratory of the First Affiliated Hospital of Soochow University (Suzhou, China). Of course, informed consent was obtained from each patient who donated their brain tissues for the research purpose. Six normal brain samples were obtained from people with traumatic brain injury, for whom a partial resection of brain tissues was conducted for decreasing the intracranial pressure. Brain tumors included 30 women and 31 men. The tumor group consists of 32 cases of grade II (26 astrocytoma, 6 oligodendroglioma), 13 cases of grade III (11 anaplastic astrocytoma, 2 anaplastic ependymoma), and 16 cases of grade IV (glioblastoma), according to the 2007 WHO classification system. The ages of people at surgical resection were 40.62 ± 15.64 years for grade II and 47.89 ± 15.21 years for grade III and grade IV. All human samples were used in accordance with the policies of the institutional review board of the First Affiliated Hospital of Soochow University.

### Cell cultures and treatments

The human U87 and U251 glioma cell lines were purchased from the Cell Bank Type Culture Collection of the Chinese Academy of Sciences (Shanghai, China). Cells were grown in Dulbecco’s modified Eagle’s medium (DMEM; Hyclone, Thermo Fisher Scientific, Waltham, MA, USA) supplemented with 10 % fetal bovine serum (FBS; Gibco, Invitrogen, Carlsbad, CA, USA).

Cells were treated with 100 nmol/ml NVP-BEZ235 (Cayman Chemical, Ann Arbor, MI, USA) for 6 h for the cell proliferation trial and 6 h for the Western blot analysis. Cells treated in parallel with the same amount of dimethyl sulfoxide (DMSO) served as controls.

### Oligonucleotide transfection

For the miR-199a-3p over-expression, cells were transfected with 100 nmol⁄L of miR-199a-3p mimics, which are small, chemically modified double-strand RNA molecules that are designed to mimic endogenous mature miRNAs. The transfection rates in two human glioma cell lines, U87 and U251, were determined by green fluorescence and flow cytometry. The transfection efficiency was analyzed by flow cytometry; the results suggested that transfection efficiency were greater than 90 %. The entire transfection process was completed using Lipofectamine 2000 (Invitrogen), according to the manufacturer’s instructions.

### Quantitative real-time PCR analysis

All of the RNA from cells and tissues was isolated using TRIzol reagent (Invitrogen) for both messenger RNA (mRNA) and miRNA analyses. For the analysis of the miR-199a-3p expression, quantitative real-time PCR (qRT-PCR) analyses were carried out using the All-in-One miRNA qRT-PCR Detection Kit (GeneCopoeia, Rockville, MD, USA), according to the manufacturer’s instructions (LightCycler 480 Roche). U6 was regarded as the internal control (miR-199a-3p primer ID, hsmq-0715, Catalog#HmiRQP0289; U6 primer ID, hsRNAU6, Catalog#HmiRQP9001; LightCycler 480 Roche). Relative levels of mTOR, P70S6K, 4EBP1, and RHOA mRNAs were examined using SYBR green real-time quantitative RT-PCR (qRT-PCR) (LightCycler480 Roche, Switzerland) and were normalized to levels of GAPDH mRNA. Relative expression was calculated using the 2^−△△CT^ method. All qRT-PCR analyses were carried out in triplicate, and the data are presented as mean and standard deviation.

### Flow cytometric analysis

Glioma cell lines (U87, U251) were cultured in 6-well plates before transfecting with the negative control oligonucleotide or mimics of miR-199a-3p. Cells were collected and fixed in 70 % cold ethanol at −4 °C for one night. Cells underwent propidium staining (Beyotime Institute of Biotechnology, Shanghai, China), and cell cycles were analyzed by flow cytometry. The detection of apoptotic cells was performed by using the PE Annexin V Apoptosis Detection Kit I (BD Biosciences, Franklin, NJ, USA). U87 and U251 were collected 72 h after transfection. Cells were washed twice with cold PBS and then resuspended in 1× binding buffer at a concentration of 1 × 10^6^ cells/ml. Then, 5 μl of 7-AAD and 5 μl of PE Annexin V were added to the culture tube with 100 μl of cell suspension. Cells were incubated for 15 min in the dark at room temperature (RT), and 400 μl 1× binding buffer was added to each tube, which was analyzed by flow cytometry within 1 h. All analyses were repeated three times.

### CCK-8 assay

The proliferation of miR-199a-3p was detected by a cell counting kit (CCK-8, Dojindo, China). The cells were transfected with a negative control oligonucleotide, or mimics of miR-199a-3p were seeded in 96-well plates at a density of 2000 cells/well. Following 6, 24, 48, and 72 h of incubation, subsequently, CCK-8 was added with 10 μl per well and incubated 2 h again, after which the absorbance at 450 nm was measured (Thermo, MA, USA). The absorption of OD was calculated as the mean ± SD of six determinations per sample, in three separate experiments.

### In vitro invasion assay

U87 and U251 cells transfected with the negative control or miR-199a-3p mimic oligonucleotides were cultured for 48 h, and cells were suspended and transferred to 200 μl solution on the top of Matrigel-coated invasion chambers (24-well insert, 8-μm pore size; BD Biosciences) with serum-free DMEM. Then, 750-μl DMEM with 0.05 % FBS was added to the lower chamber. After 72 h had elapsed, cells above the matrigel were removed and cells were fixed with 4 % formaldehyde for 10 min and stained with 0.1 % crystal violet for 20 to 30 min. Invading cells were counted in six randomly selected sites under a microscope.

### Western blot

Cells were collected after culturing for 72 h and mechanically lysed in the mixture lysis buffer for the Western blot and phenylmethylsulfonyl fluoride (PMSF) (Beyotime Institute of Biotechnology). The protein concentration was detected by a bicinchoninic acid (BCA) protein assay kit (Beyotime Institute of Biotechnology). The primary antibodies used were anti-mTOR, anti-p-AKT, anti-P70S6K, anti-p-P70S6K, anti-4E-BP1, anti-p-4E-BP1, anti-RHOA, anti-matrix metallopeptidase 2 (MMP2), anti-Caspase-3, and anti-GAPDH (CST, MA, USA). Protein samples were separated by 6 to 15 % SDS-PAGE and transferred to a nitrocellulose membrane, which was blocked with 5 % bovine serum albumin (BSA) (Amresco, Solon, OH, USA) for 1 h at RT. Membranes were incubated with primary antibodies overnight at 4 °C. The membranes were washed and incubated with HRP-conjugated secondary antibodies (Beyotime Institute of Biotechnology) for 2 h. The signal was detected using enhanced chemiluminescence (ECL) (Thermo).

### Immunofluorescence analysis

The cells were fixed with 4 % paraformaldehyde for 30 min and blocked with BSA (Amresco) for 30 min. The cells were then incubated with primary antibodies for the detection of p-mTOR (diluted 1:50), p-AKT (diluted 1:200), and p-4E-BP1 (diluted 1:200) at 4 °C overnight and incubated with tetramethylrhodamine isothiocyanate-labeled secondary antibody (diluted 1:400) at 37 °C for 1 h. Finally, cells were stained with DAPI, and photos were taken with a fluorescence microscope (OLYMPUS BX50/BXFLA/DP70; Olympus Co., Japan).

### Immunohistochemistry analysis

U87 and U251 cells were incubated with the primary antibody anti-Ki-67 (CST). Sections seeded with cells were incubated according to manufacturer’s instructions offered by the Cell and Tissue Staining Kit HRP-DAB system (R&D Systems, Minneapolis, MN, USA).

### Statistical analysis

Statistical analyses were conducted with SPSS 13.0 software (SPSS Inc., Chicago, IL, USA). Differences between groups were tested using a Student’s *t* test or ANOVA. Reported *P* values were two-tailed and were considered statistically significant when *P* < 0.05.

## Results

### Expression of miR-199a-3p, mTOR, and its downstream effector molecules in glioma samples

**Fig. 1:**
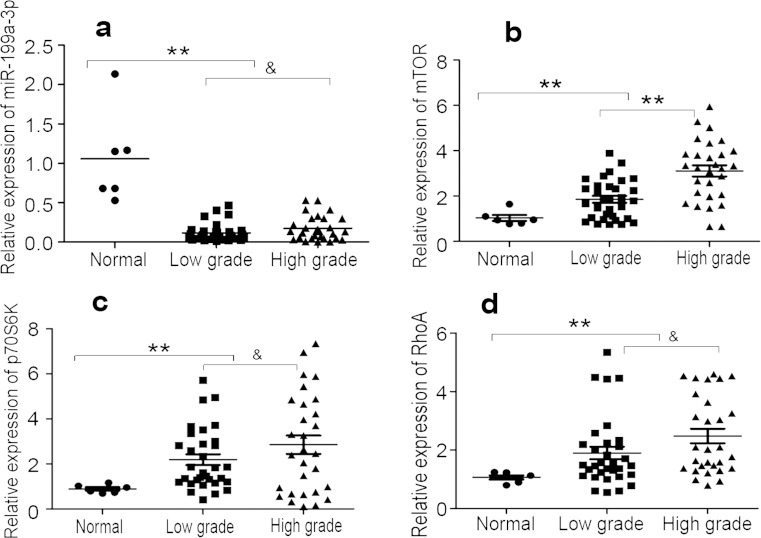
The expression of miR-199a-3p, mTOR, p70S6K, and RhoA in glioma samples and normal brain tissue were assessed by qPCR. Data were reported as 2^−△△CT^. **a** miR-199a-3p in human glioma samples compared with normal brain tissues (*asterisks*, *P* < 0.01), in low grade (grade II) compared with high grade (grade III, grade IV) (*ampersand*, *P* > 0.05). **b** mTOR in glioma samples compared with normal brain tissues (*asterisks*, *P* < 0.01), mTOR expression with the increasing degree of glioma (low-grade glioma vs. high-grade glioma; *asterisks*, *P* < 0.01). **c**, **d** The expression of p70S6K and RhoA in glioma samples compared with normal brain tissues (*asterisks*, *P* < 0.01) and in low grade and high grade (ampersand, *P* > 0.05)

**Fig. 2:**
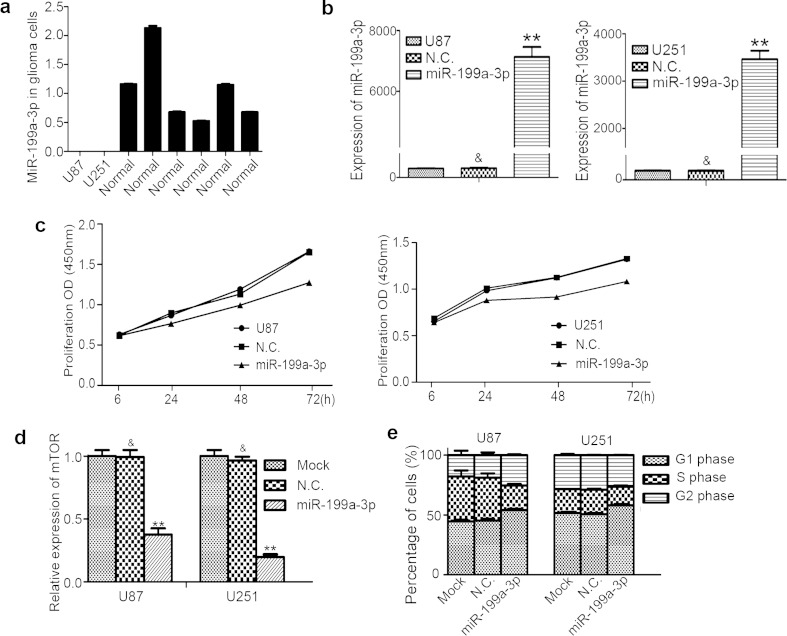
**a** The expression of miR-199a-3p in U87 and U251 compared with six normal brain tissues (*asterisks*, *P* < 0.01). **b** The expression of miR-199a-3p in cells transfected with miR-199a-3p (*asterisks*, *P* < 0.01). **c** Cellular proliferation was detected in U87 and U251 by cell counting kit (CCK-8) after 6, 24, 48, and 72 h of incubation. Data were reported as means ± SD (*asterisks*, *P* < 0.01), compared with cells transfected with miR-199a-3p mimic negative control or untransfected cells. **d** mTOR in U87 and U251 with miR-199a-3p over-expression compared with miR-199a-3p mimic negative control (*asterisks*, *P* < 0.01). **e** Cell cycle modulation was restrained resulting in G1 phase increase (*asterisks*, *P* < 0.01) and S phase decrease (*asterisk*, *P* < 0.05)

To gain further insight into the expression of miR-199a-3p in glioma samples and normal brain tissues, quantitative polymerase chain reaction (qPCR) was conducted in 6 normal brain tissues and 61 glioma samples. Results showed that the expression of miR-199a-3p was lower in glioma samples than normal brain tissues (*P* < 0.01; Fig. 1a), and there was no significant difference between low-grade glioma (grade II) and high-grade (grade III, grade IV) glioma (*P* > 0.05; Fig. 1a). The expression of mTOR in glioma samples was higher combined with normal brain tissues and increased with the increasing degree of malignancy in glioma (low-grade glioma vs. high-grade glioma, *P* < 0.01; Fig. 1b). In addition, RhoA and p70S6k expressions in glioma samples were higher than normal brain tissues (*P* < 0.01; Fig. 1c, d) and had no significant association in low-grade and high-grade glioma samples (*P* > 0.05; Fig. 1c, d). The expression of miR-199a-3p in U87 and U251 cell lines was very low (*P* < 0.01; Fig. 2a).

### Up-regulation of miR-199a-3p suppresses proliferation in glioma cells

**Fig. 3:**
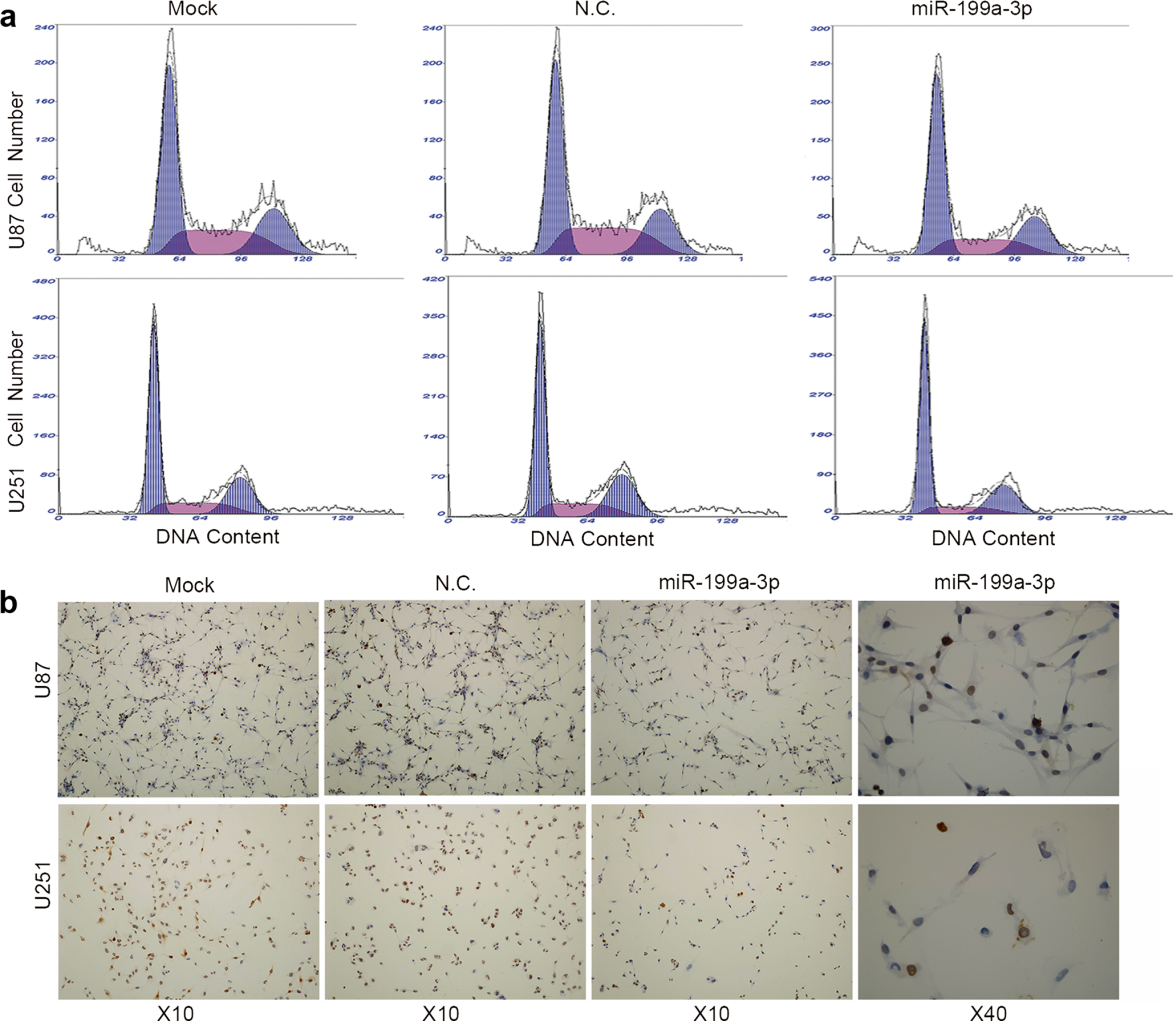
Cellular proliferation was evaluated by cell cycle by flow cytometry analysis and detecting the expression of Ki-67 immunohistochemistry analysis. **a** Cell cycle modulation was restrained compared with the negative control group. **b** The expression of Ki-67 was reduced in cells transfected with miR-199a-3p

Glioma has a poor prognosis; it grows as rapidly as any other malignancy. We conducted cellular proliferation assays in U87 and U251 cells to assess whether the up-regulation of miR-199a-3p has a suppressive effect on the growth of glioma cells. The expression of miR-199a-3p was higher after cells were transfected with miR-199a-3p mimics (*P* < 0.01; Fig. 2b). As a result, we found that cell proliferation was distinctly decreased in glioma cells after transfection with miR-199a-3p (*P* < 0.01; Fig. 2c). To gain further insight into cell cycle modulation, flow cytometry analysis was performed in U87 and U251 cells. Results revealed that the G1 phase (*P* < 0.01; Figs. 2e and 3a) was increased, and the S phase was decreased (*P* < 0.05; Figs. 2e and 3a) in cells with the over-expression of miR-199a-3p. Immunohistochemistry in U87 and U251 cells transfected with miR-199a-3p showed reduced Ki-67, compared with negative control glioma cells (Fig. 3b). Hence, the data revealed that miR-199a-3p up-regulation inhibited cell proliferation in glioma.

### Mir-199a-3p had no effect on invasion and apoptosis capability

**Fig. 4:**
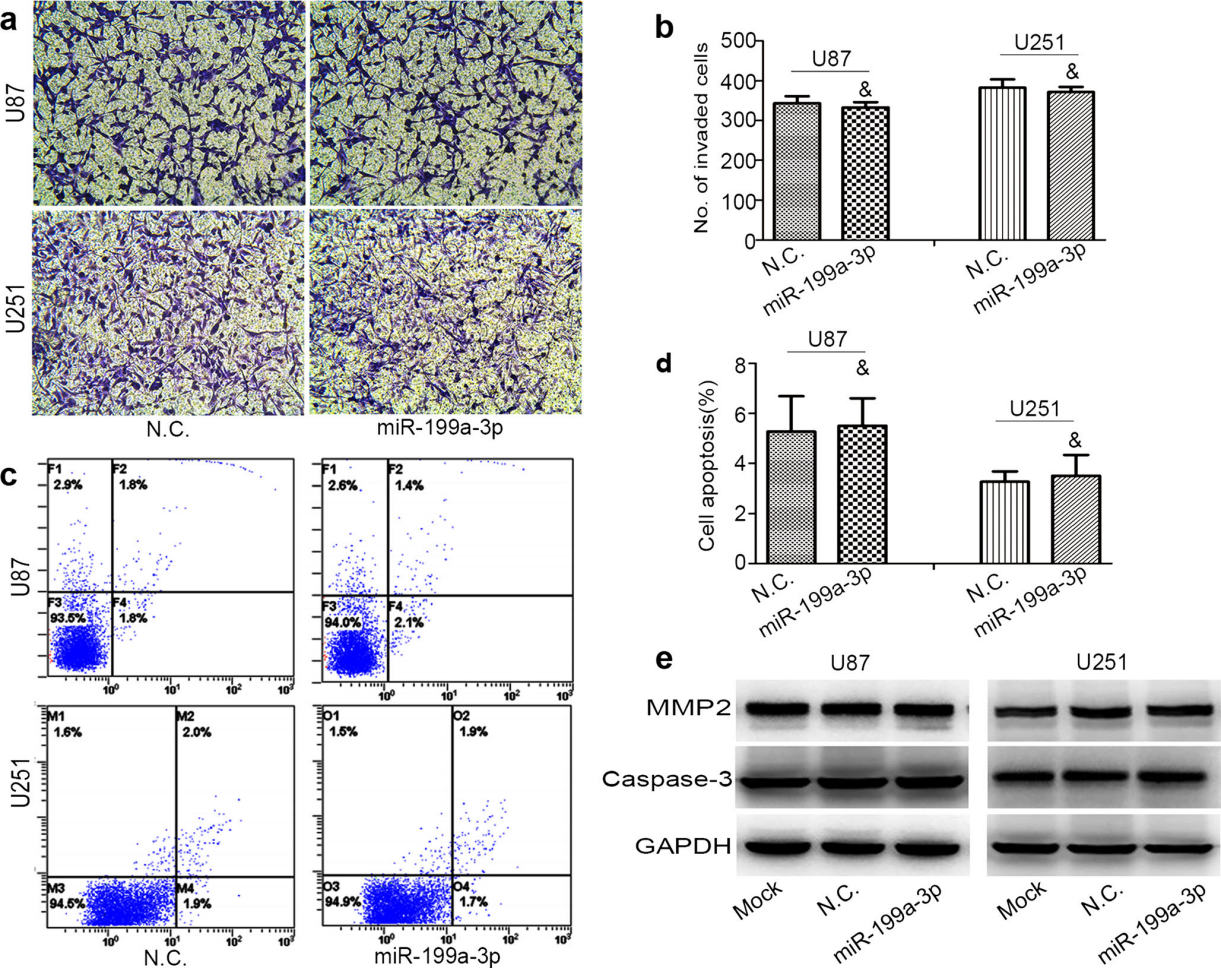
The invasion and apoptosis were assessed and compared with miR-199a-3p mimic negative control. **a**, **b** Invasion ability was conducted by transwell invasion system. The number of invasive cells was counted (*ampersand*, *P* > 0.05). **c**, **d** Apoptosis capability was detected by flow cytometry analysis (*ampersand*, *P* > 0.05). **e** The expression of MMP2 and caspase-3 were conducted (*ampersand*, *P* > 0.05)

To determine whether the suppression of cellular proliferation was due to decreased invasion capability or increased apoptosis by the over-expression of miR-199a-3p, we carried out a cell invasion assay and apoptosis assay in vitro. At the same time, we detected the expression of MMP2 and caspase-3, which were related to invasion and apoptosis capability, by Western blot analysis. Results indicated that no significant difference was found between glioma cells’ over-expression of miR-199a-3p and those with negative control oligonucleotide (*P* > 0.05; Fig. 4).

### Relationship between miR-199a-3p and mTOR

**Fig. 5:**
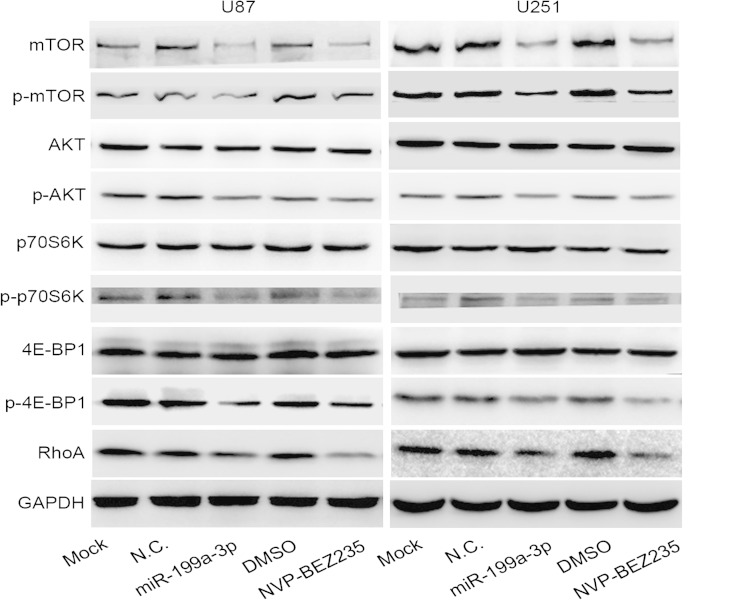
The expression of mTOR, p-mTOR, AKT, p-AKT, p70S6K, p-p70S6K, 4E-BP1, p-4E-BP1, RhoA, and GAPDH in different groups were detected by Western blot analysis

**Fig. 6:**
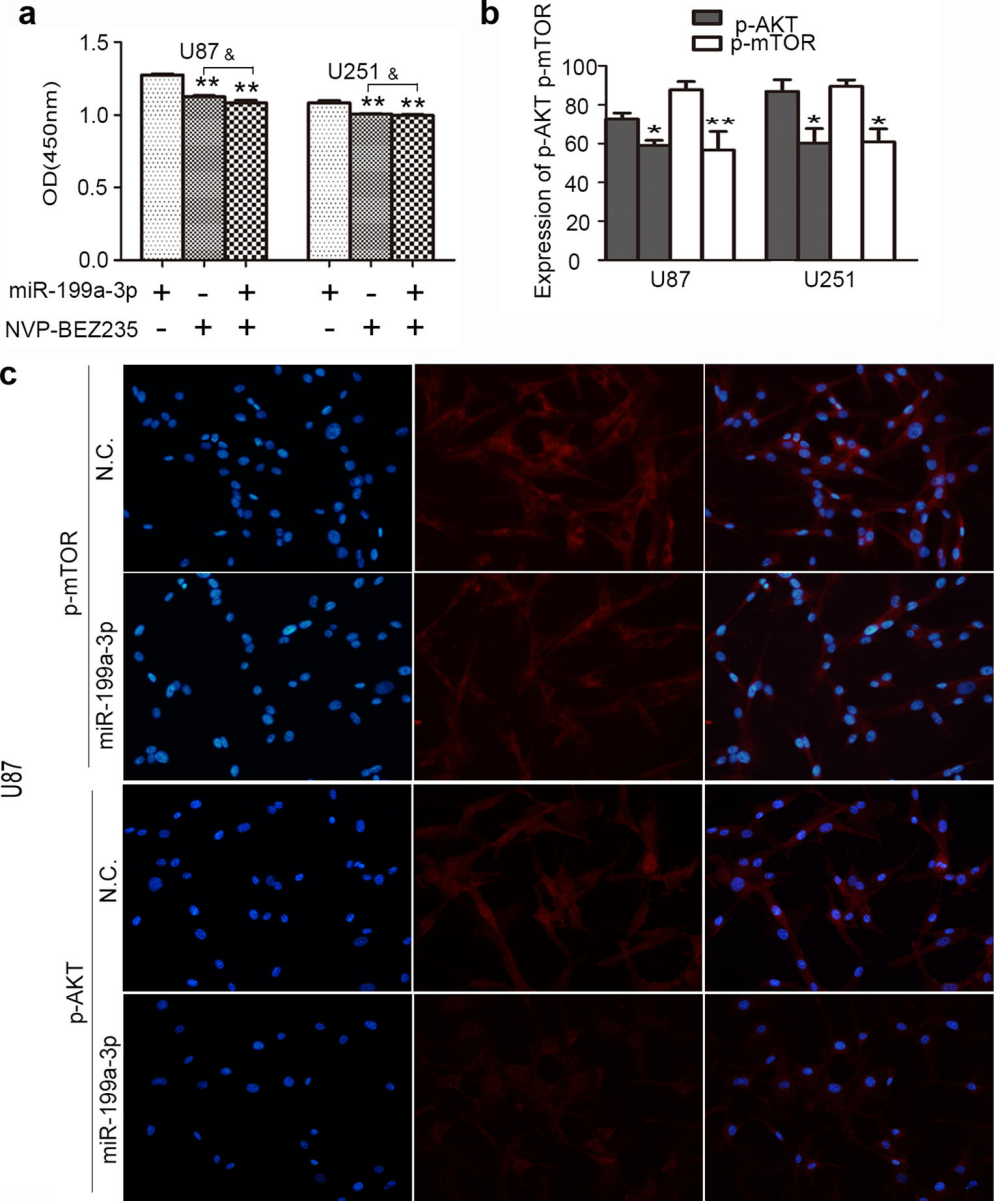
**a** Cellular proliferation was assessed after 72 h of incubation. Cells were treated with NVP-BEZ235 of 100 nmol/L for 24 h and then transfected further with miR-199a-3p (*ampersand*, *P* > 0.05), when compared with cells treated with NVP-BEZ235 only. **b**, **c** Immunofluorescence analysis showed that the expression of mTOR (*asterisk*, *P* < 0.05) and p-AKT (*asterisks*, *P* < 0.01) was apparently inhibited in cells by the up-regulation of miR-199a-3p

**Fig. 7:**
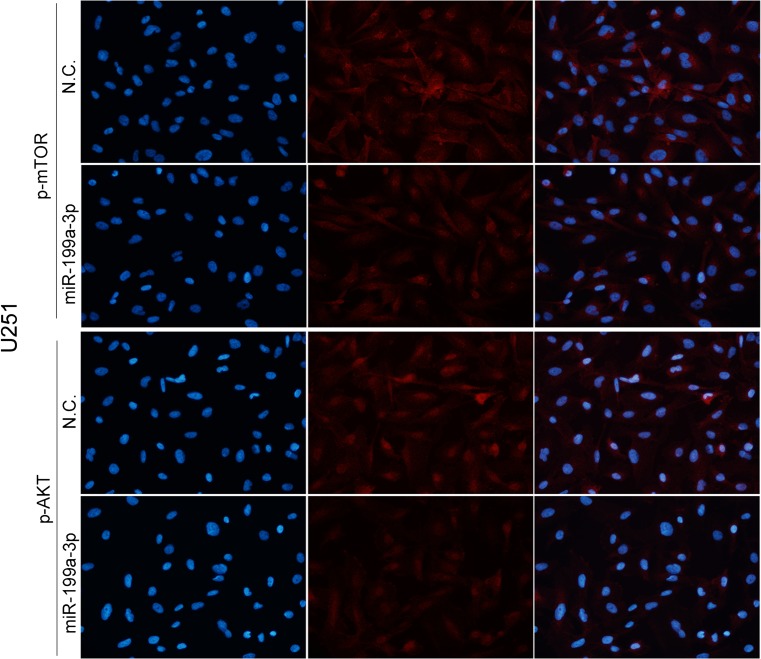
Immunofluorescence analysis showed that the expression of mTOR and p-AKT (*asterisk*, *P* < 0.05) was apparently inhibited in cells by the up-regulation of miR-199a-3p in U251

Dates from the miRBase database and many studies [15, 17–19] had proved that mTOR was targeted by miR-199a-3p. AIQurashi et al.[20] reported that miR-199a-3p acted a role in tumor suppression by targeting mTORC1 and mTORC2. Moreover, the expression of mTOR in U87 and U51 cell lines obviously decreased after transfection with miR-199a-3p (*P* < 0.01; Fig. 2d). In addition, to estimate the effect of miR-199a-3p on AKT/mTOR, we also found mTOR protein levels and the phosphorylation of mTOR, AKT, p70S6K, and 4E-BP1 decreased in miR-199a-3p groups by Western blot analysis and immunofluorescence staining (*P* < 0.01; Figs. 5, 6, and 7). We further assessed the biological significance of miR-199a-3p in the regulation of mTOR expression in glioma cells. Cells treated with NVP-BEZ235 to inhibit mTOR activity and further transfected with miR-199a-3p did not have a significant difference in inhibiting cell proliferation (*P* > 0.05; Fig. 6a).

## Discussion

Varieties of previous powerful evidence revealed that many miRNAs were abnormally expressed in human glioma samples or cell lines [21–23]. Our pre-experiment has shown that hsa-miR-199a-3p is one of them. Furthermore, the dysregulation of miR-199a-3p in glioma cell lines has similar effects to a tumor suppressor gene or proto-oncogene. In our study, miR-199a-3p suppresses glioma cellular growth and proliferation via regulating AKT/mTOR signaling pathway.

MiRNAs bind to partially or fully complementary sequences of mRNA in the 3′-untranslated region (3′-UTR) to induce mRNA degradation or repress mRNA translation [24, 25], then influencing the output of many protein-coding genes [26]. For example, the down-regulation or up-regulation of miR-199a-3p has been shown to be related to cell proliferation, invasion, drug resistance, apoptosis, and other processes in some kinds of malignancy by targeting related genes. Although a variety of miRNAs in glioma has been investigated in vivo and in vitro, the research regarding miR-199a-3p in glioma is limited. Gu and colleagues [27] reported that three out of five glioma samples were significantly elevated regarding miR-199a-3p when compared to two normal brain tissues. However, in the present study, we expended the number of glioma samples and examined the expression of miR-199a-3p by real-time PCR. Results showed miR-199-3p has a lower expression in glioma samples and that there is no significant differential expression between low-grade and high-grade glioma. Tumor specificity or instability may contribute to the inconformity that comes from Gu and our team.

The mTOR is a ubiquitously expressed serine/threonine (Ser/Thr) kinase that is a crucial regulator of cell proliferation, metabolism, and other biological progresses in a lot of tumors [4, 28]. Inhibitors of mTOR, for example, NVP-BEZ235 and miRNAs, block the AKT/mTOR signaling pathway and suppress tumor cells’ growth in many malignant tumors [29–31], including glioma. Results from our study implied that miR-199a-3p plays a critical role as a kind of inhibitor of mTOR in glioma. The over-expression of miR-199a-3p had a link to a lower level of protein expression of mTOR and p-mTOR by bonding the mTOR gene and then regulating the phosphorylation status of 4E-BP1, p70S6K, and AKT in the mediation of cell growth and cell proliferation. However, the changed level of p-AKT after the inhibition of mTOR is controversial [32–34]. The argument will last; the reason is unsure but may be due to varying experimental settings between different investigators.

The invasion capability and apoptosis resistance of tumor cells have been a barrier to therapeutic strategies. Fornari et al. found that miR-199a-3p in human hepatocarcinoma cells caused an obvious decrease of invasive potential [18]. In addition, Vignot et al. confirmed that inhibiting mTOR had a relation to promoting apoptosis in some tumors [30, 35]. However, viewing the results from the present study, we tentatively propose that the up-regulation of miR-199a-3p in glioma cell lines, U87 and U251, has no significant difference in invasion and apoptosis. It seems that the effect of the cell biological function through inhibiting the expression of mTOR may vary with origins of tumors or depends on cellular characteristics of tumor cells. Besides that, miRNAs may not be an independent factor at times or target potential genes other than mTOR. Shafer et al. discovered rapamycin, one kind of inhibitor of mTOR, combined with paclitaxel-induced apoptosis, but not rapamycin alone [36].

Studies have reached an agreement with the view that tumor cell proliferation is decreased if a lower level of mTOR is obtained [37–39]. Gaining further insight into the mechanisms of miR-199a-3p cell cycle modulation, we find that miR-199a-3p targets mTOR and phosphorylates p70S6K and 4E-BP1, downstream effector molecules of mTOR, to regulate cell growth and proliferation. In addition, a decreased expression of the Ras homolog gene family, member A (RhoA), which combined with mTOR in the context of cytoskeletal regulation [40, 41], is discovered in the high miR-199a-3p group. Fornari et al.[15, 18] believed that cell cycle modulation by mTOR inhibitors eventually led to an increased G1 phase and decreased S phase. Indeed, results from our study indicated why glioma cell proliferation was limited when we elevated the expression of miR-199a-3p. In our study, we found mTOR and p-mTOR, relative protein of AKT/mTOR signaling pathway, were reduced in miR-199a-3p group. We believed miR-199a-3p suppresses the AKT/mTOR signaling pathway and cell proliferation in vitro. Evidence suggested that mTOR might be one of the targeted genes of miR-199a-3p.

Taken together, miR-199a-3p has been found dysregulated in some malignancies. Further study proved it was down-regulated in glioma samples and worked with effects similar to the tumor suppressor gene. The effective role miR-199a-3p played in glioma can be attributed to the suppression of the AKT/mTOR signaling pathway. Several decades had elapsed before therapeutic strategies were found in connection with the cure of glioma. Today, miR-199a-3p might provide another approach against glioma via targeting oncogenes.
